# Niclosamide-Modulated Apoptosis and Autophagy in Breast Cancer Cells via Phosphorylated JNK as a Common Regulator

**DOI:** 10.7150/ijms.106429

**Published:** 2025-06-23

**Authors:** Ming-Shan Chen, Tsung-Yi Chen, Shu-Hsin Chen, Shew-Meei Sheu

**Affiliations:** 1 Department of Anesthesiology, Ditmanson Medical Foundation Chia-Yi Christian Hospital, Chia-Yi City 60002, Taiwan.; 2 Department of Medical Research, Ditmanson Medical Foundation Chia-Yi Christian Hospital, Chia-Yi City 60002, Taiwan.; 3 Department of Biotechnology, Asia University, 41354 Taichung City, Taiwan.

**Keywords:** niclosamide, apoptosis, autophagy, breast cancer, JNK

## Abstract

Autophagy plays critical pro-survival and pro-apoptotic roles in regulating breast cancer death. Niclosamide is a U.S. FDA-approved drug that is used for parasite treatment. Exposure to niclosamide causes apoptosis in several different types of cancer cells, whereas its ability to regulate autophagy remains limited, especially in breast cancer. In this study, we evaluated the relative mechanism by which niclosamide regulates apoptosis and autophagy in breast cancer cells. We found that niclosamide induced G0/G1 cell cycle arrest and apoptosis in MCF-7 and T-47D cells. It also caused the turnover of microtubule-associated protein 1 light chain 3 (LC3-II), an autophagy marker, and arrested autophagosome maturation. Niclosamide-induced apoptosis was inhibited by an autophagy initiator (3-methyladenine) but significantly enhanced by chloroquine, an autophagy blocker. Both Jun-amino-terminal kinase (JNK) and reactive oxygen species (ROS) inhibitors decreased LC3-II accumulation and niclosamide-induced apoptosis. However, the ROS inhibitor reduced the expression of niclosamide-activated p-JNK in MCF-7 cells but not in T-47D cells. In conclusion, blocking autophagy is cytotoxic and promotes niclosamide-induced apoptosis. Phosphorylated JNK is identified for the first time as a common regulator of niclosamide-induced autophagy and apoptosis, acting through ROS-dependent or ROS-independent pathways.

## Introduction

Breast cancer is the most common cancer among women globally, with more than 685,000 deaths reported in 2020 1. Advancements in early diagnostic techniques and treatment strategies have significantly improved survival rates 2, but cancer metastasis, the emergence of drug resistance, and the occurrence of severe side effects remain challenges for clinical practice.

Based on gene expression profiles from tumor cDNA microarrays, breast cancer can be classified into five or six intrinsic subtypes 3, 4. The most common subtype, luminal A, typically presents strong luminal gene signatures, along with the presence of estrogen receptor (ER) and progesterone receptor. However, intrinsic and acquired drug resistance and the limited effectiveness of adjuvant chemotherapy in improving recurrence-free survival, particularly in node-positive patients, remain major challenges in the treatment of luminal A breast cancer 5, 6.

Niclosamide is a U.S. FDA-approved anthelmintic drug that has been used for the eradication of parasitic infection for approximately half a century. In addition to its conventional use, several studies have demonstrated its potential anticancer effects against a variety of cancer types, including breast, prostate, colorectal, lung, and ovarian cancers 7-11. These effects can occur through the suppression of STAT3 8, inactivation of the NF-κB pathway and generation of reactive oxygen species (ROS) 12, inhibition of the mTORC1 pathway 13 or inhibition of the Wnt/β-catenin signaling pathway 14. The potential impact of niclosamide on breast cancer cells and its associated molecular mechanism have not yet been completely investigated.

Autophagy is a physiological process that degrades and transports damaged organelles and misfolded proteins to the lysosome. It can play either pro-survival or pro-apoptotic roles during breast cancer progression 15, 16. Antiestrogen resistance in ER^+^ breast cancer has been linked to autophagy 17. One study reported that niclosamide modulates autophagy in breast cancer stem cells 18. Modulating autophagy to promote apoptosis may offer a promising strategy for cancer therapy. In the present study, we used the ER^+^ luminal A subtype cells (MCF-7 and T-47D) to determine the mechanism by which niclosamide inhibits cell proliferation, including cell cycle arrest, apoptosis, and autophagy-regulated apoptosis.

## Materials and Methods

### Cell culture

Human breast cancer cell lines, specifically those with the luminal A subtype (MCF-7 and T-47D), were purchased from the Bioresource Collection and Research Center in Hsinchu, Taiwan. MCF-7 cells were maintained in RPMI 1640 medium (Gibco BRL, Grand Island, NY, USA) supplemented with 8% fetal bovine serum (FBS), while T-47D cells were cultured in RPMI 1640 medium supplemented with 8% FBS, 1.5 g/L sodium bicarbonate, 4.5 g/L glucose, 10 mM HEPES, 1.0 mM sodium pyruvate, and 0.2 I.U. bovine insulin per milliliter. Both cell lines were cultured in a humidified atmosphere containing 5% CO_2_ at 37 ˚C.

### Reagents and antibodies

Niclosamide and *N*-acetyl-L-cysteine (NAC) were obtained from Sigma-Aldrich (St. Louis, MO, USA), while rapamycin was purchased from LC Laboratories (Woburn, MA, USA). 3-methyladenine (3-MA) was collected from Adooq Bioscience (Irvine, CA, USA). Antibodies against poly (ADP-ribose) polymerase (PARP) (#9542), phospho-SAPK/JNK (Thr183/Tyr185) (#4668), JNK2 (#9258), phospho-p44/42 MAPK (Erk1/2) (Thr202/Tyr204) (#4377), p44/42 MAPK (Erk1/2) (#4695), phospho-p38 MAPK (Thr180/Tyr182) (#9211), p38 MAPK (#9212), phospho-STAT3 (Tyr705) (#9145), STAT3 (#4904) and β-catenin (#8480) were purchased from Cell Signaling Technology, Inc. (Beverly, MA, USA). A rabbit polyclonal antibody against the autophagosomal marker protein, microtubule-associated protein 1 light chain 3 (LC3), was acquired from Abcepta, Inc. (San Diego, CA, USA). Furthermore, a mouse monoclonal antibody against the sequestosome 1 protein, also known as p62, was obtained from Santa Cruz Biotechnology (Santa Cruz, CA, USA). An anti-glyceraldehyde-3-phosphate dehydrogenase (GAPDH) monoclonal antibody was obtained from Taiclone (Taipei, Taiwan).

### Cell viability

Cells were plated (5×10^3^ cells/well) in 96-well plates and allowed to incubate overnight. The cells were subsequently incubated with a concentration series of niclosamide (0, 1, 2.5, 5, and 10 μM) for 24, 48 or 72 hours. Cell viability was assessed using Cell Counting Kit-8 (Sigma-Aldrich), the addition of WST-8 solution to cells were then incubated for two hours. Finally, the absorbance at 450 nm was recorded with a Model 680 microplate reader (Bio-Rad Laboratories, Inc., Hercules, CA, USA), using a reference wavelength of 655 nm. The absorbance of cells treated with niclosamide was divided by that of the solvent control (DMSO) to determine the percentage of viable cells.

### Colony formation assay

Colony-forming cells were plated (2×10^3^ cells/well) in 12-well plates, incubated overnight and then treated with the indicated concentrations of niclosamide. During incubation for 6-12 days, the culture medium was replenished every three days. Colonies were finally fixed with 4% paraformaldehyde and visualized by staining with 1% crystal violet (Sigma-Aldrich), after which digital images were captured. The quantification of the colonies was performed using the Alpha Innotech imaging system (Alphatron Asia Pte. Ltd., Singapore).

### Cell cycle and apoptosis analyses

Cells (1×10^6^) were allowed to adhere to a 10-cm dish overnight, followed by a 24-hour period of serum starvation in preparation for cell cycle analysis. Concentrations of 2 and 5 µM were selected based on the IC_50_ for MCF-7 and T-47D cells being approximately 2 µM and 5 µM resulting in greater inhibition of cell viability. Treatment durations of 48 and 72 hours were chosen because significant inhibition was observed at these time points; 24-hour treatments (1-10 µM) did not effectively inhibit cell viability. After treatment with solvent (0.1% DMSO) or niclosamide (2 and 5 μM) for 48 and 72 h, the cells were detached with trypsin and centrifuged at 2,000 rpm for five minutes. The resulting cell pellets were dissociated into single-cell suspensions, fixed with methanol and stored at 4 °C. The cells were then stained with 40 µg/ml propidium iodide (PI) (Sigma-Aldrich) containing RNase (50 µg/ml) for 30 min in the dark at room temperature (RT), and a FACScan flow cytometer (Becton Dickinson, San Diego, CA, USA) was used to analyze the cell cycle distribution. The DNA content was further evaluated using ModFit LT 5.0 software. For the apoptosis assay, cells treated with or without niclosamide (2 and 5 μM) for 48 and 72 h were collected and labeled using an Annexin V-fluorescein isothiocyanate (FITC) apoptosis detection kit (Strong Biotech, Taipei, Taiwan) and PI staining. The apoptotic cells were further assessed using a FACScan flow cytometer (Becton Dickinson).

### Measuring autophagy with a RFP-GFP-LC3B Kit

Cells were cultured at a density of 1×10^4^ in a 35*12-mm glass dish (Alpha Plus Scientific Corp.) overnight. The PremoTM Autophagy Tandem Sensor Red Fluorescent Protein (RFP)-Green Fluorescent Protein (GFP)-LC3B Kit (Life Technologies, Carlsbad, CA, USA) was used to track autophagy progression, following the manufacturer's instructions. First, the cells were exposed to 4.5 μl of BacMam reagent containing RFP-GFP-LC3B DNA overnight, and then the specified concentrations of niclosamide were added before incubation for 48 hours. The autophagic flux inducer, rapamycin (30 μM), was used as a positive control. Hoechst 33342 (1 μg/mL) was used to label the nuclei for 20 minutes in the dark. The cells were then washed with 1× phosphate-buffered saline, and images were captured using a Zeiss LSM800 laser scanning confocal microscope (Carl Zeiss Microscopy GmbH, Jena, Germany).

### Western blotting

To prepare the cell lysates for western blotting, the cells were dissolved in M-PERTM mammalian protein extraction reagent (Thermo Fisher Scientific Inc., Rockford, IL, USA) supplemented with 0.1% protease inhibitor cocktail. Each sample was loaded with an equal amount of protein (40 μg) and fractionated via sodium dodecyl sulfate-polyacrylamide gel electrophoresis, followed by transfer to polyvinylidene fluoride membranes (EMD Millipore Corporation, Billerica, MA, USA). The target proteins on the membranes were incubated with primary antibodies (1:1000) overnight at 4 °C and subsequently incubated with horseradish peroxidase-conjugated secondary antibodies (1:10000) at RT for one hour. Band signals were detected by Immobilon Western Chemiluminescent HRP Substrate (EMD Millipore Corporation) and visualized using a BioSpectrum® imaging system (UVP).

### Statistical analysis

The data used in this study are presented as the means ± standard deviations (SDs). Statistical significance was determined using one-way ANOVA with a post hoc test and Bonferroni correction to compare multiple groups or with an independent t test to compare two groups using SPSS (Windows version 21), and *p* values of **<** 0.05 were considered to indicate statistical significance.

## Results

### Niclosamide inhibited cell proliferation

**Figure 1:**
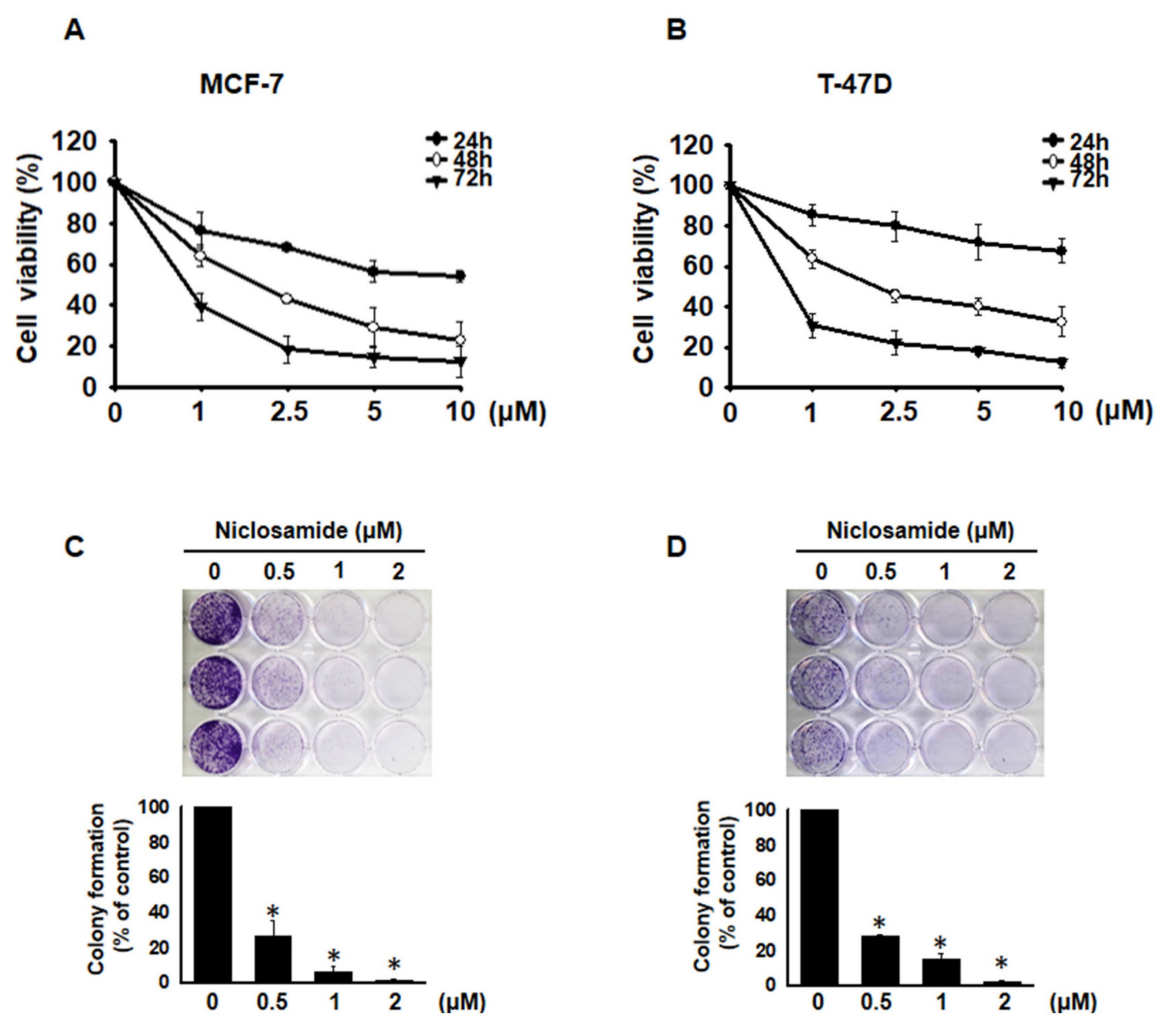
Niclosamide-induced inhibition of MCF-7 and T-47D cell proliferation. MCF-7 (A) and T-47D (B) cells were incubated with various concentrations of niclosamide (1-10 μM) for 24, 48 and 72 hours. A CCK-8 assay was used to detect and calculate cell viability. Cell viability is expressed as the means ± SDs of three independent experiments. Colony formation assays were performed on MCF-7 (C) and T-47D (D) cells treated with or without 0.5-2 μM niclosamide for 6-12 days. Colony formation was visualized using crystal violet staining. The results from three independent experiments are presented as the means ± SDs compared with the 0.1% DMSO control. Statistical significance was assessed using one-way ANOVA followed by Bonferroni correction (p < 0.05). An asterisk (*) denotes a significant difference from the DMSO control.

To investigate whether niclosamide inhibits the proliferation of MCF-7 and T-47D cells, we performed a CCK-8 assay. After treatment with niclosamide at concentrations ranging from 1 to 10 μM for 24, 48 and 72 h, niclosamide decreased cell viability in a dose- and time-dependent manner (Figs. 1A and 1B). The IC_50_ values were 2.0 μM for MCF-7 cells and 2.1 μM for T-47D cells at 48 h. Additionally, colony formation assays revealed that niclosamide dose-dependently decreased colony numbers in both cell lines (Figs. 1C and 1D). These findings indicate that niclosamide effectively inhibits breast cancer cell proliferation *in vitro*.

### Niclosamide induced G0/G1 cell cycle arrest and apoptosis

**Figure 2:**
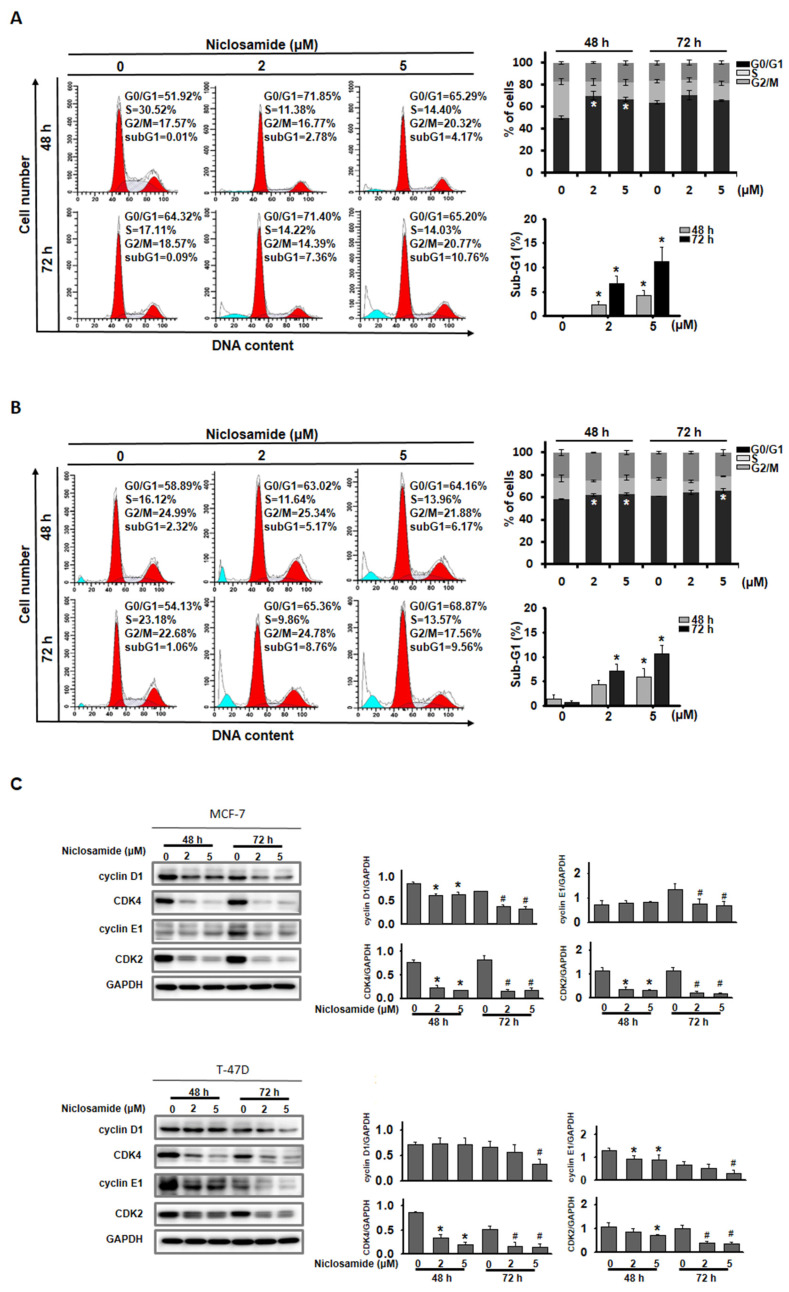
Niclosamide induced G0/G1 cell cycle arrest. MCF-7 (A) and T-47D (B) cells were treated with niclosamide at concentrations of 2 and 5 μM for 48 and 72 h. Cell cycle distribution was subsequently analyzed using flow cytometry. The graphs show representative data on the cell cycle distribution and mean percentages of each cell cycle phase from three independent experiments displayed in the histograms. The proportions of sub-G1 cells following treatment with niclosamide for 48 and 72 h are presented in the accompanying histogram. (C) Lysates of niclosamide-treated MCF-7 and T-47D cells (48 and 72 h) were subjected to western blotting to detect cell cycle regulatory proteins. An asterisk (*) and a hash (#) indicate significant differences (* p < 0.05 vs. 48 h DMSO; # p < 0.05 vs. 72 h DMSO), determined using one-way ANOVA followed by Bonferroni correction.

To examine the potential mechanism by which niclosamide inhibits cell proliferation, its effects on the cell cycle and the induction of apoptosis were assessed using flow cytometry. The percentage of MCF-7 and T-47D cells in the G0/G1 phase significantly increased after treatment with 2 and 5 μM niclosamide for 48 h (Figs. 2A and 2B), and this increase continued for 72 h in T-47D cells treated with 5 μM niclosamide. Regulatory proteins associated with the G0/G1 phase were further analyzed by western blotting (Fig. 2C). Niclosamide treatment decreased the expression of cyclin D1, CDK2 and CDK4 in MCF-7 cells at 48 and 72 h, which was consistent with the expression patterns in T-47D cells after 72 h of treatment. Cyclin E1 expression was repressed in MCF-7 cells at 72 h and decreased in T-47D cells at 48 and 72 h in the presence of niclosamide. Furthermore, significant sub-G1 accumulation was detected in both cell types after treatment with 2 and 5 μM niclosamide for 48 and 72 h (Figs. 2A and 2B).

### Niclosamide induced apoptosis and arrested autophagic flux

**Figure 3:**
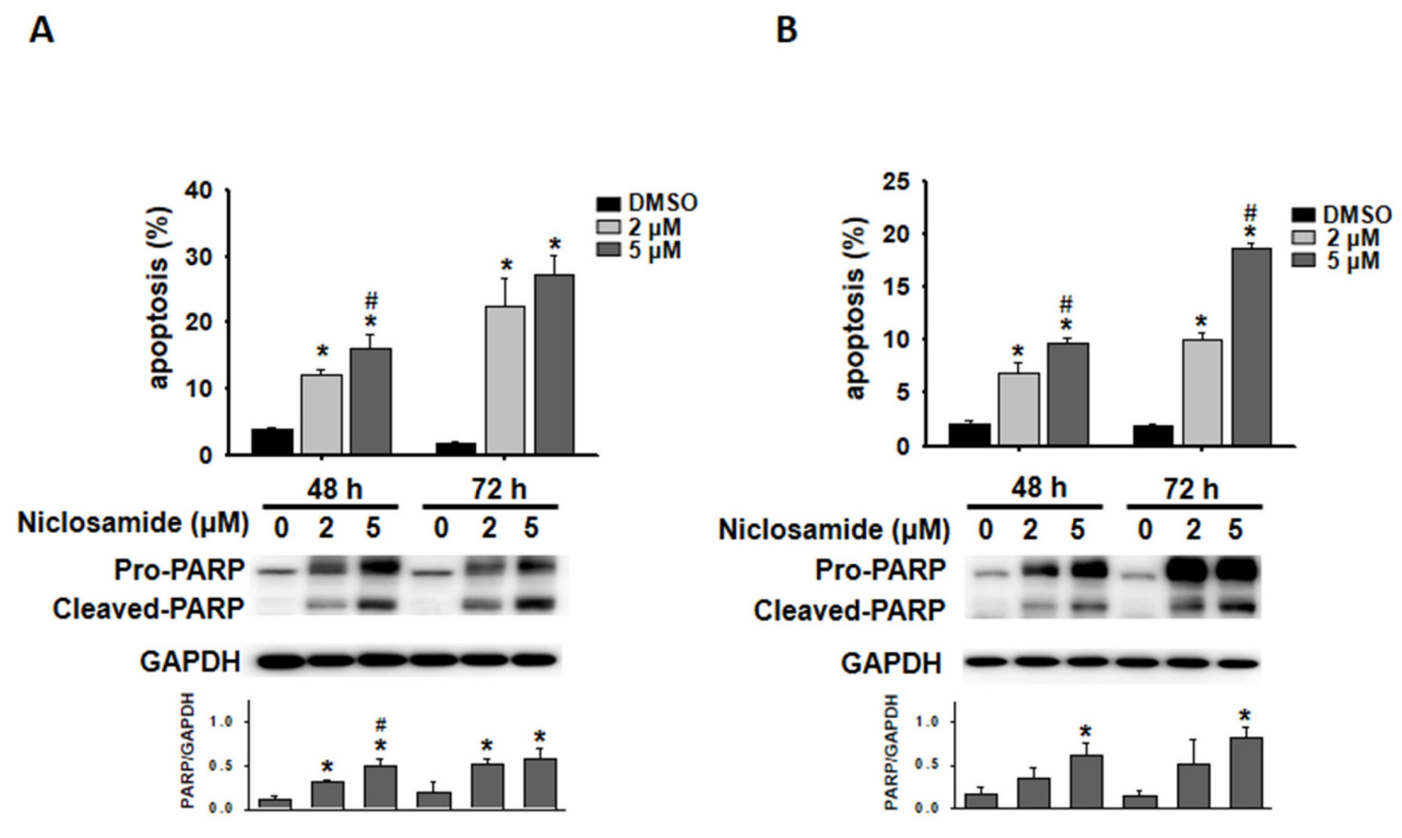
Niclosamide induced apoptosis in breast cancer cells. MCF-7 (A) and T-47D (B) cells were treated with niclosamide (2 and 5 μM) for 48 and 72 h. Niclosamide-induced apoptosis was visualized using Annexin V-FITC/PI staining and flow cytometry. Three separate apoptosis experiments were conducted for each cell line, and the results are expressed as the means ± SDs. Western blotting was used to examine the increased expression of cleaved-PARP. The band intensity of cleaved-PARP relative to that of GAPDH was calculated and presented in a histogram. An asterisk (*) indicates a significant difference compared with the control, and a hash (#) indicates a significant difference compared with the niclosamide treatment. Statistical analysis was performed using one-way ANOVA followed by Bonferroni correction.

Niclosamide-treated cells were visualized with Annexin V-FITC/PI staining, and the proportion of apoptotic cells was further analyzed via flow cytometry. Niclosamide induced apoptosis in a dose- and time-dependent manner in both MCF-7 and T-47D cells (Figs. 3A and 3B). Western blotting revealed that niclosamide obviously increased the expression of cleaved-PARP, a caspase-dependent apoptosis marker, compared with that in control cells.

**Figure 4:**
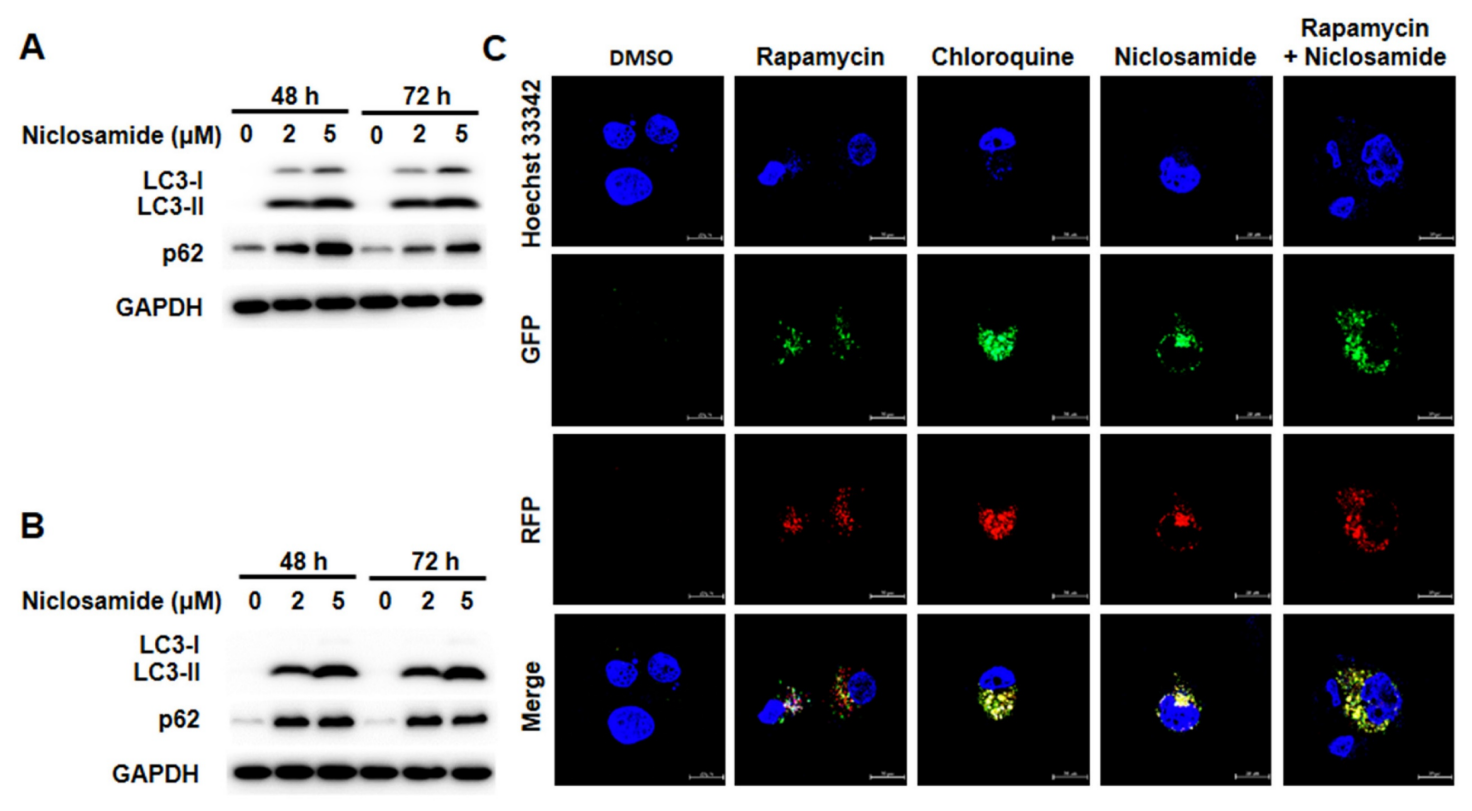
Niclosamide promoted the accumulation of LC3-II in a dose-dependent manner and blocked autophagic flux. MCF-7(A) and T-47D (B) cells were treated with niclosamide (2 and 5 μM) for 48 and 72 h. Autophagy markers, including LC3-II accumulation and p62 expression, were identified via western blotting, using GAPDH as a loading control. The data represent three independent experiments. (C) MCF-7 cells were transduced with tandem RFP-GFP-LC3B to visualize the fluorescent autophagosomes and autophagolysosomes. Cells expressing RFP-GFP-LC3B were treated with niclosamide (5 μM), chloroquine (50 μM), rapamycin (30 μM), or a combination of 2 drugs. In the merged images, yellow or orange dots (RFP-GFP-LC3B) indicate autophagosomes, whereas red dots (RFP-LC3B) indicate autophagolysosomes, as the green fluorescence is quenched in acidic lysosomes. Images were captured using a 63× oil lens, and the scale bar is 20 μm.

Autophagic flux is commonly assessed by analyzing the turnover of the autophagy marker LC3 (LC3-II) via western blotting. To determine whether niclosamide modulates autophagy, we found that niclosamide increased LC3-II turnover in a dose-dependent manner in MCF-7 and T-47D cells (Figs. 4A and 4B). The Autophagy Sensor Kit from Life Technologies was further employed to visualize the stages of autophagic flux, revealing autophagosome fusion with lysosomes to form autolysosomes via RFP-GFP-LC3B fluorescent proteins. Initially, LC3-II located in autophagosomes (neutral pH) exhibited both GFP (green) and RFP (red) signals, appearing as yellow/orange dots. Upon fusion with lysosomes, the pH-sensitive GFP fluorescence was subsequently quenched, resulting in the autolysosomes appearing as red dots. Niclosamide induced the accumulation of LC3-II in autophagosomes (yellow/orange), similar to chloroquine, an autophagy blocker that disrupts autophagosome maturation. Induction of autophagic flux with rapamycin can be also inhibited by niclosamide (Fig. 4C). These results indicate that niclosamide has the ability to arrest autophagic flux.

### Niclosamide-induced apoptosis was enhanced by chloroquine

**Figure 5:**
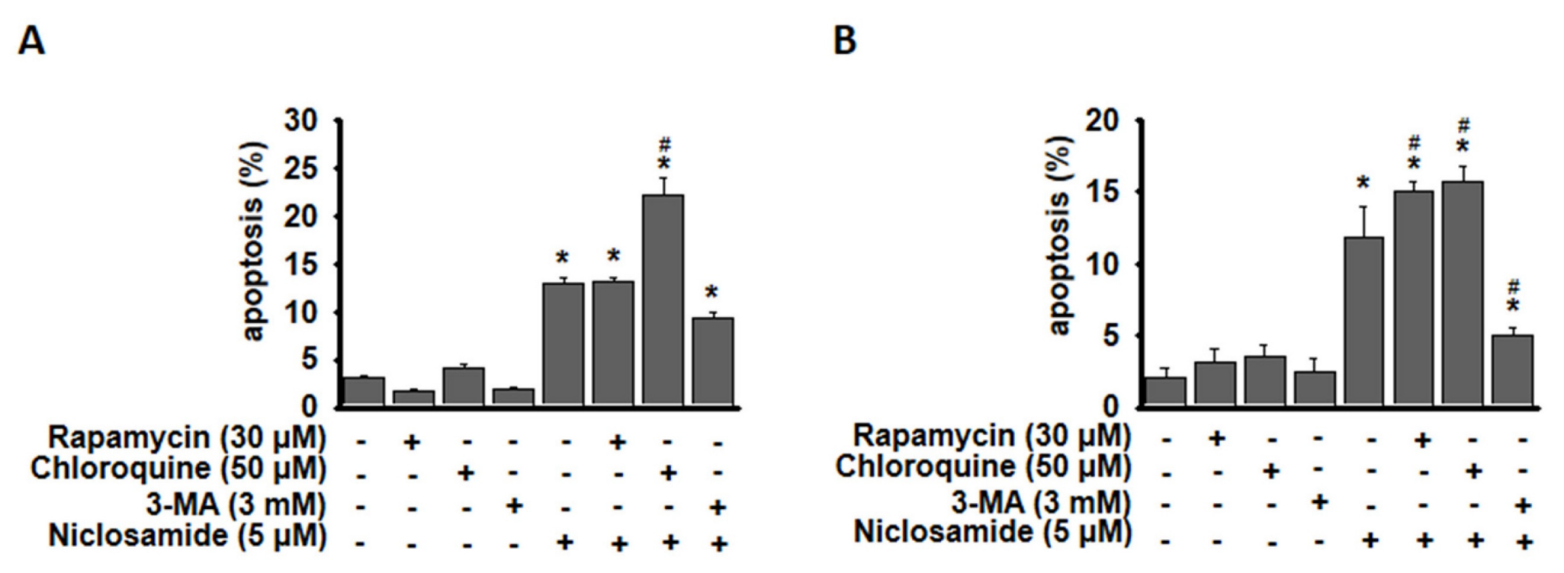
Blocking autophagy enhanced niclosamide-induced apoptosis. MCF-7 (A) and T-47D (B) cells were preincubated with or without chloroquine, rapamycin or 3-MA for 2 h, followed by incubation with 5 μM niclosamide for 48 hours, with or without the indicated reagents. Apoptosis was assessed using flow cytometry. The data were collected from three independent experiments and are presented as the means ± SDs. An asterisk (*) indicates a significant difference compared with the control (p < 0.05), and a hash (#) indicates a significant difference compared with niclosamide treatment, determined by one-way ANOVA followed by Bonferroni correction.

To determine whether niclosamide-induced apoptosis in MCF-7 and T-47D cells is related to autophagy, we induced or inhibited autophagy using rapamycin or 3-MA, a specific autophagosome formation inhibitor (Fig. 5). The combined treatment of niclosamide with rapamycin enhanced niclosamide-induced apoptosis in T-47D cells, whereas 3-MA significantly inhibited niclosamide-induced apoptosis in both cells. Niclosamide-induced apoptosis was significantly enhanced by chloroquine in MCF-7 and T-47D cells.

### Phosphorylated JNK involved in niclosamide-mediated autophagy and apoptosis

**Figure 6:**
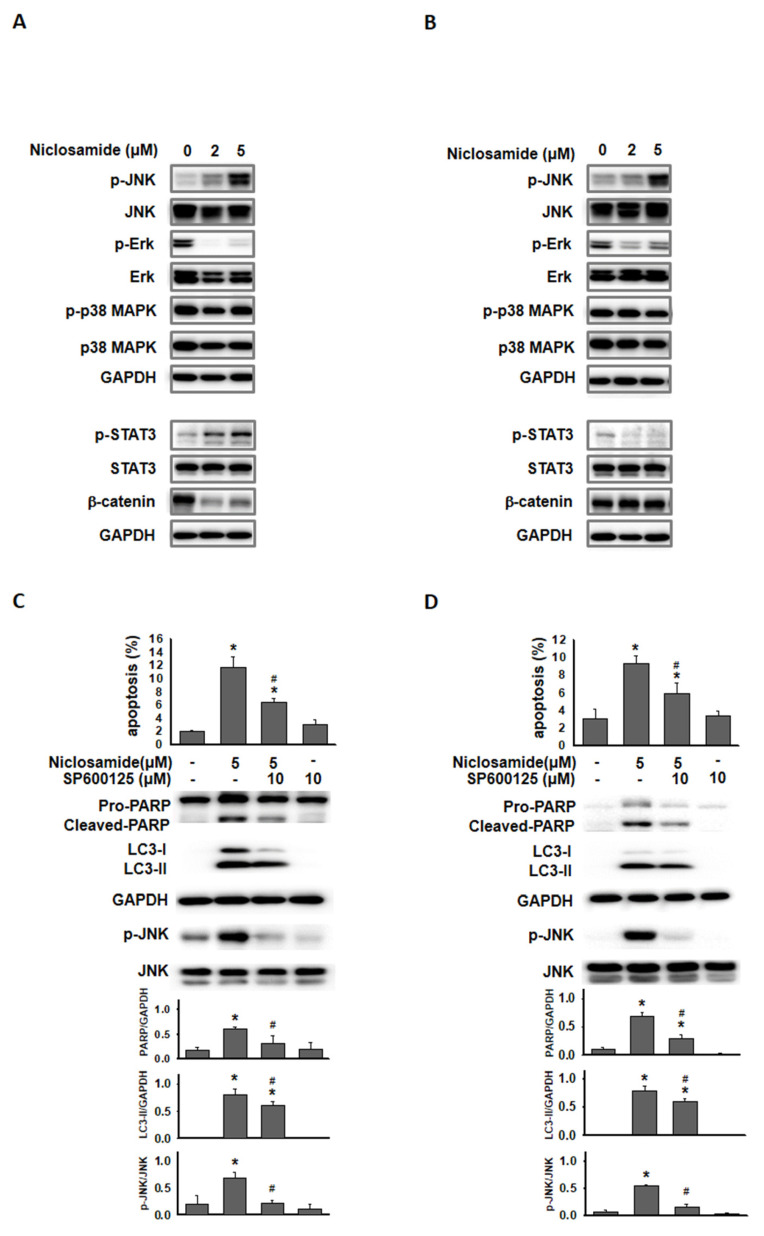

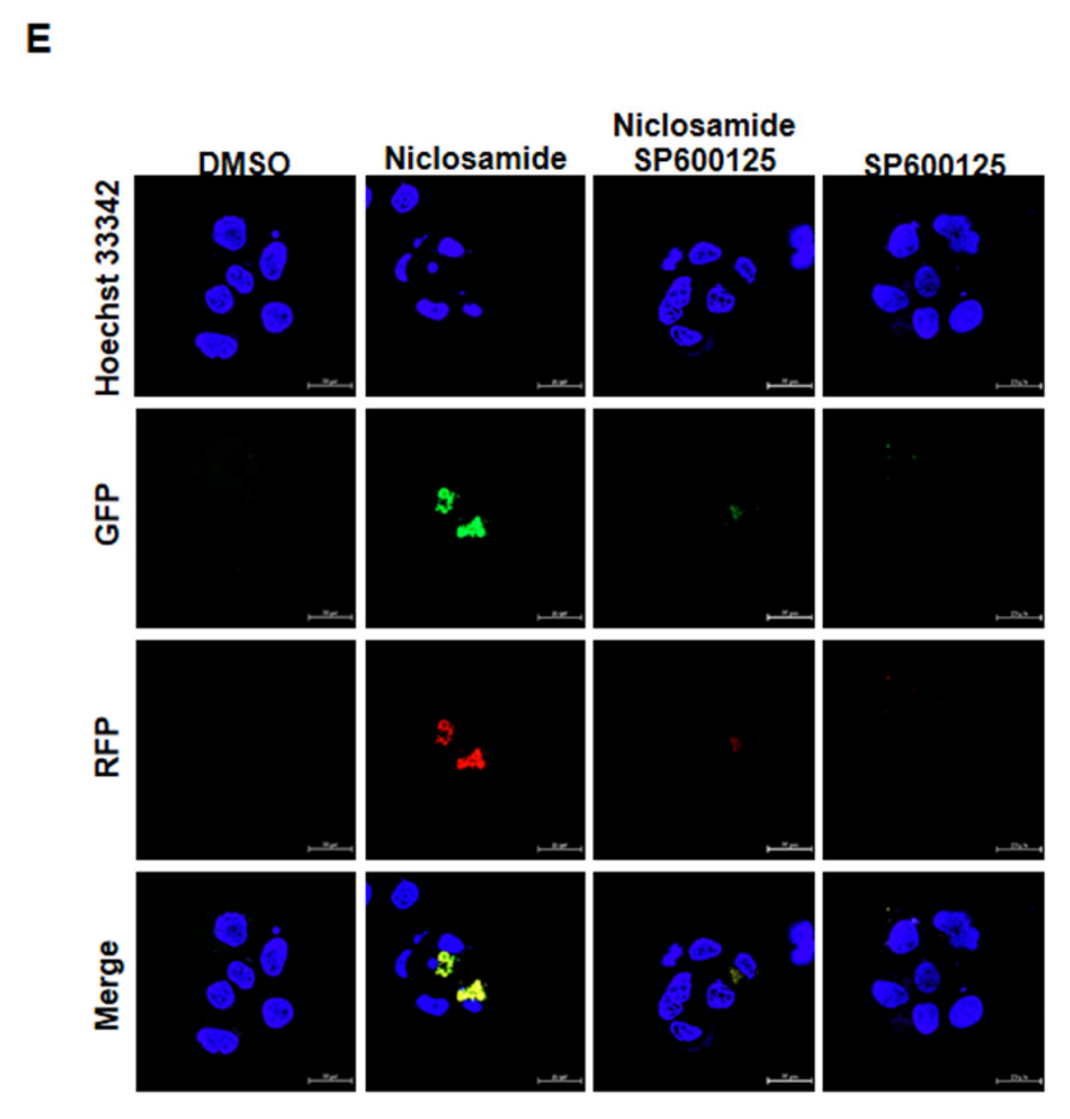
** Inhibition of p-JNK reduced niclosamide-induced apoptosis and LC3-II expression.** MCF-7 (A) and T-47D (B) cells were treated with niclosamide for 48 h. Cell pellets were collected for the detection of the indicated molecules by western blotting. MCF-7 (C) and T-47D (D) cells were preincubated with or without a JNK inhibitor (SP600125) for 2 h. Then, niclosamide was added to the cells for 48 h in the presence or absence of SP600125. Apoptotic cells were labeled with Annexin V-FITC/PI staining, and the percentage of positive cells was analyzed via flow cytometry. Western blotting was used to detect the expression of cleaved-PARP, LC3-I, LC3-II, p-JNK, JNK, and GAPDH. Protein expression ratios from three independent experiments were calculated relative to GAPDH or total JNK, depending on the target protein. An asterisk (*) indicates a significant difference compared with the control (p < 0.05), and a hash (#) indicates a significant difference compared with niclosamide treatment, determined by one-way ANOVA followed by Bonferroni correction. (E) MCF-7 cells expressing RFP-GFP-LC3B were treated with niclosamide (5 μM), SP600125 (10 μM) or a combination of both drugs. In the merged images, autophagosomes are indicated as yellow dots (RFP-GFP-LC3B). Images were captured using a 63× oil lens, and the scale bar is 20 μm.

Jun-amino-terminal kinase (JNK) signals are known to mediate autophagy induction 19, 20. We also investigated ERK, p38 MAPK and signaling molecules (β-catenin and p-STAT3) that have been demonstrated to be regulated by niclosamide 9, 21. As shown in Figs. 6A and 6B, p-ERK expression was obviously reduced by niclosamide. The expression of phosphorylated STAT3 and β-catenin exhibited opposite patterns in niclosamide-treated MCF-7 and T-47D cells. The only molecule that was markedly increased in a dose-dependent manner in both cells was p-JNK. To investigate the role of p-JNK in niclosamide-mediated autophagy and apoptosis, niclosamide was applied with the JNK inhibitor SP600125, and reduced expression of LC3-II and cleaved-PARP was observed (Figs. 6C and 6D). The combined treatment induced a lower percentage of apoptosis compared to niclosamide alone in MCF-7 and T-47D cells. Additionally, niclosamide-arrested autophagosome maturation (yellow or orange dots) was reversed by the addition of SP600125 (Fig. 6E). These findings suggest that p-JNK is a common pathway regulating both apoptosis and autophagy in these cells.

### ROS-dependent and ROS-independent effects on JNK activation

**Figure 7:**
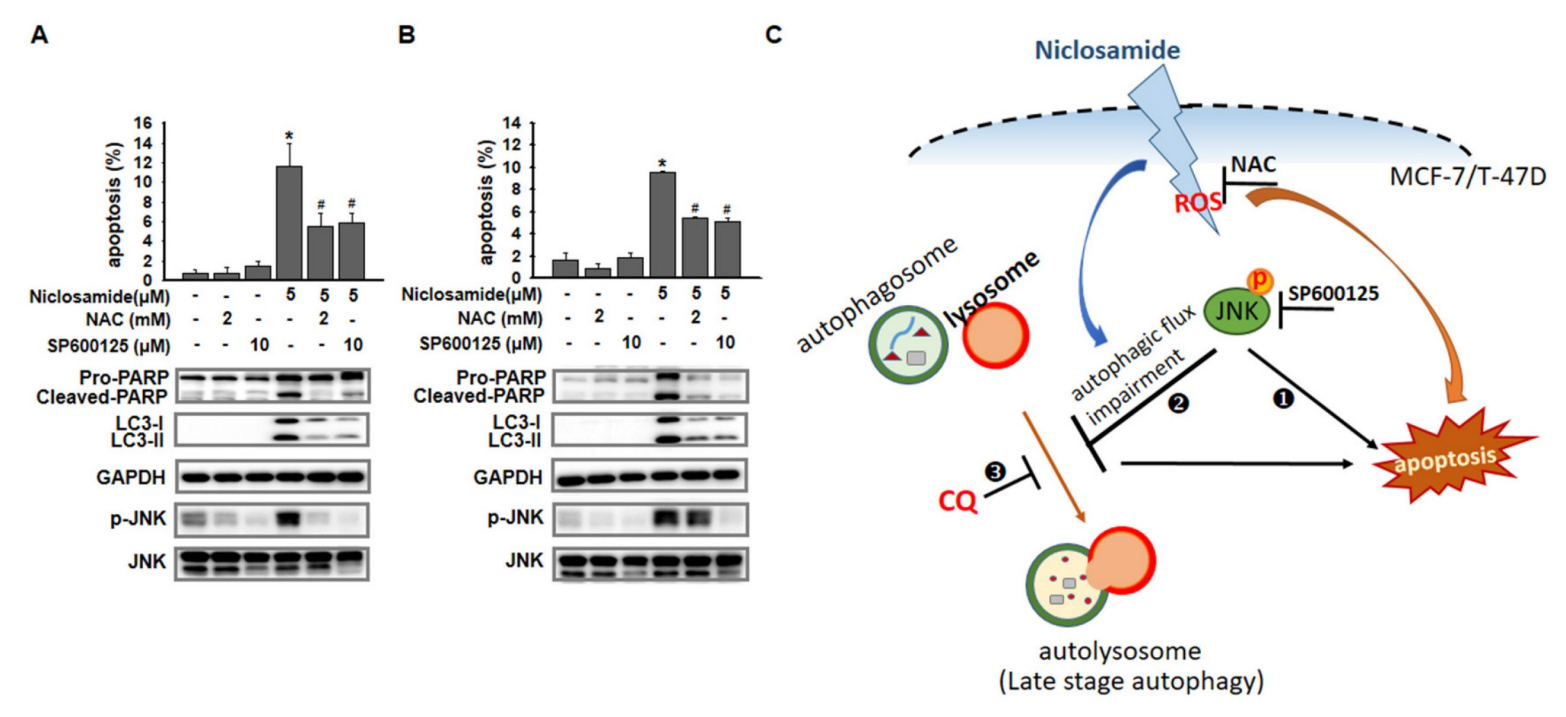
** p-JNK was activated via ROS-dependent or ROS-independent pathways.** MCF-7 (A) and T-47D (B) cells were preincubated with or without a JNK (SP600125) or ROS (NAC) inhibitor for 2 h. After treatment with the indicated reagents for 48 h, cell pellets were collected for analysis of apoptosis by flow cytometry and for detection of the targeted molecules by western blotting. (C) Niclosamide induced apoptosis and inhibited autophagosome maturation via common JNK activation in MCF-7 and T-47D cells. The role of JNK activation in niclosamide-induced apoptosis (❶) and niclosamide-inhibited autophagic flux (❷) was demonstrated by the addition of a JNK inhibitor (SP600125). Niclosamide combined with chloroquine (CQ, ❸) further enhanced apoptosis, indicating that blocking autophagy enhanced its cytotoxic effect. Phosphorylated JNK can be activated through ROS-dependent (MCF-7 cells) or ROS-independent (T-47D cells) pathways. Treatment with a ROS (NAC) or a JNK (SP600125) inhibitor partially reversed niclosamide-induced apoptosis.

ROS regulate key cell signaling pathways that trigger the initiation of apoptosis 22. We used NAC to inhibit ROS and investigated the apoptotic proportions of niclosamide-treated cells (Figs. 7A and 7B). Compared with the niclosamide treatment alone, the combination of niclosamide and NAC significantly suppressed apoptosis and decreased p-JNK expression in MCF-7 cells, whereas p-JNK expression was maintained in T-47D cells.

## Discussion

Autophagy appears to play a dual role in luminal breast cancer, acting as either a protector or a promoter of cell death. It can influence the initiation, proliferation, and progression of tumors. Niclosamide, an FDA-approved drug for treating tapeworm infections, has been demonstrated to have anticancer effects. We investigated whether niclosamide could modulate autophagy and further cause breast cancer death. We found that niclosamide induced G0/G1 cell cycle arrest, significantly induced apoptosis and blocked autophagy in both MCF-7 and T-47D cells. Combined treatment with 3-MA further suppressed niclosamide-induced apoptosis, whereas the addition of chloroquine enhanced niclosamide-induced apoptosis by blocking autophagy. The JNK inhibitor SP600125 reduced the accumulation of LC3-II and inhibited niclosamide-induced apoptosis, indicating that p-JNK is critical for the interaction between niclosamide-induced apoptosis and autophagy.

The IC_50_ value of niclosamide in various cancer cells is approximately 2 μM after 48 hours of treatment, with a significant decrease in cell viability observed at 48 hours and beyond 23, 24. These findings are consistent with the results shown in Fig. 1. The antiproliferation mechanism includes the induction of cell cycle arrest and cell death. In osteosarcoma, niclosamide induced S and G2/M cell cycle arrest 25. Fig. 2 shows that niclosamide induced G0/G1 cell cycle arrest. The same effect has also been observed in cells of other types of cancer, including adrenocortical carcinoma, esophageal squamous cell carcinoma, head and neck squamous cell carcinoma, and oral squamous cell carcinoma 26-29. These results indicate that niclosamide dominantly induces G0/G1 cell cycle arrest. The transition from the G0/G1 to S phases is regulated by cyclin D-CDK4/6 and cyclin E-CDK2 complexes 26, 30. The results in Fig. 2C support previous findings that CDK2 and CDK4 levels are significantly reduced in G0/G1-arrested HL-60 cells treated with isoindigo 5′-Br 31, and oral squamous cell carcinoma cells treated with niclosamide 26. Cyclin D1, but not cyclin E, was decreased in isoindigo 5′-Br treated HL-60 cells 31, whereas both cyclins D1 and E were consistently inhibited in esophageal cancer cells 28, MCF-7 and T-47D breast cancer cells treated with niclosamide (Fig. 2C). These findings suggest that niclosamide induces G0/G1 phase arrest by downregulating genes encoding key cell cycle regulatory proteins.

Niclosamide can suppress the proliferation of human cancers by inducing apoptosis, as observed in osteosarcoma, non-small cell lung cancer and T‑cell acute lymphoblastic leukemia *in vitro* 23, 25, 32. In breast cancer, niclosamide-induced apoptosis was observed in MDA-MB-231 and T-47D cells 21. Our present study demonstrated that niclosamide could induce apoptosis in a dose-dependent (2 and 5 μM) and time-dependent (48 and 72 h) manner in MCF-7 and T-47D cells (Fig. 3). A previous study revealed that niclosamide (10 μM) could trigger autophagy within 4 hours in MCF-7 and HeLa/GFP-LC3 cells 13, 33. Hansen *et al.* reported that while niclosamide (0.5 μM) increased the formation of autophagosomes in MCF-7 cells at later time points during 16 h of investigation, each cell still had more autophagolysosomes (red puncta) than autophagosomes (yellow puncta) 34. We revealed that niclosamide treatment (5 μM) for 48 h promoted LC3-II accumulation and arrested autophagosome maturation (Fig. 4), resulting in cells having more autophagosomes than autophagolysosomes. We provide evidence that longer exposure (48 h) to 5 μM niclosamide activated apoptosis but blocked autophagy.

Apoptosis and autophagy may share common regulatory factors 15. Induction of autophagy may cause sensitization or resistance to apoptosis. Huang *et al.* reported that niclosamide (1-2 μM) induced autophagy and apoptosis in T‑cell acute lymphoblastic leukemia cells at 24 h 32. Niclosamide (2.5 μM) induced autophagy (24 h) and delayed apoptosis (after 48 h) in human non-small cell lung cancer (NSCLC) cells 23. In NSCLC cells, 3-MA enhances niclosamide-induced apoptosis 23, but in breast cancer cells, 3-MA reduced it (Fig. 5). However, disrupting autophagosome maturation with chloroquine increased niclosamide-induced apoptosis (Fig. 5), suggesting that blocking autophagy promotes cytotoxicity and enhances niclosamide's pro-apoptotic effects in breast cancer cells. We used 3-MA and chloroquine to inhibit autophagy either at the initiation stage or upon fusion with lysosomes, leading to opposite effects on autophagy-related apoptosis. Inhibition of autophagy at a late stage by chloroquine increased niclosamide-induced apoptosis (Fig. 5). Combining niclosamide with an appropriate autophagy inhibitor is crucial for regulating autophagy-related apoptosis, implying a strategy to overcome possible treatment failure in ER^+^ breast cancer. Potential limitations of niclosamide-based combination therapy include optimizing dosage and timing, ensuring efficient drug delivery, confirming autophagy modulation *in vivo*, and managing chloroquine-related side effects.

Niclosamide-induced apoptosis has been reported to occur through the downregulation of STAT3 pathways in colorectal cancer (CRC) cells 9 or the inhibition of NF-kB downstream targets in acute myelogenous leukemia stem cells 12. Moreover, niclosamide could stimulate autophagy and inhibit the mTORC1 signaling pathway within 4 hours in MCF-7 cells 13, whereas the mTOR and NF-κB pathways were not targeted by niclosamide in CRC cell lines 14. These findings suggest that niclosamide targets multiple intracellular signaling pathways involved in apoptosis and autophagy, which may be unique for cells of different cancers. In this study, we demonstrate for the first time that niclosamide-induced apoptosis and LC3-II accumulation are partially mediated by p-JNK (Fig. 6), highlighting the crucial regulatory role of p-JNK in both apoptosis and autophagy signaling pathways in ER^+^ breast cancer cells. Moreover, p-JNK is uniquely activated through ROS-dependent or ROS-independent pathways in MCF-7 and T-47D breast cancer cells (Figs. 7A and 7B). Ye *et al.* 24 reported that the intraperitoneal administration of 20 mg/kg/day niclosamide suppressed breast tumor growth without toxicity, increased the number of cleaved caspase-3-positive cells, and reduced lung metastasis in mice. These findings support niclosamide as a potential therapeutic candidate for breast cancer.

## Conclusions

In summary, niclosamide exerted anti-proliferative effects in MCF-7 and T-47D cells by causing G0/G1 cell cycle arrest, inducing apoptosis, and blocking autophagy. Blocking autophagy with chloroquine enhanced niclosamide-mediated apoptosis. Activation of p-JNK regulates apoptosis induction and autophagy arrest through ROS-dependent or ROS-independent pathways (Fig. 7C). Combining niclosamide with chloroquine offers a potential strategy to enhance treatment of ER^+^ breast cancer, although its clinical application still warrant further investigation.

## Abbreviations

JNK: Jun-amino-terminal kinase
ER: estrogen receptor
ROS: reactive oxygen species
FBS: fetal bovine serum
NAC: *N*-acetyl-L-cysteine
3-MA: 3-methyladenine
PARP: poly (ADP-ribose) polymerase
LC3: microtubule-associated protein 1 light chain 3
GAPDH: glyceraldehyde-3-phosphate dehydrogenase
PI: propidium iodide
RT: room temperature
FITC: fluorescein isothiocyanate
RFP: red fluorescent protein
GFP: green fluorescent protein
SD: standard deviation
NSCLC: non-small cell lung cancer
CRC: colorectal cancer
